# Health Risk Assessment of Heavy Metals in Indoor Household Dust in Urban and Rural Areas of Chiang Mai and Lamphun Provinces, Thailand

**DOI:** 10.3390/toxics11121018

**Published:** 2023-12-14

**Authors:** Kawinwut Somsunun, Tippawan Prapamontol, Todsabhorn Kuanpan, Teetawat Santijitpakdee, Kanyapak Kohsuwan, Natwasan Jeytawan, Nathaporn Thongjan

**Affiliations:** 1 Environment and Health Research Group, Research Institute for Health Sciences (RIHES), Chiang Mai University, Chiang Mai 50200, Thailand; kawinwut_s@cmu.ac.th (K.S.); todkuanpan@gmail.com (T.K.); teetawat_san@cmu.ac.th (T.S.); kanyapak_koh@cmu.ac.th (K.K.); natwasan_jeytawan@cmu.ac.th (N.J.); nathaporn_t@cmu.ac.th (N.T.); 2 PhD Degree Program in Environmental Science, Environmental Science Research Center, Faculty of Science, Chiang University, Chiang Mai 50200, Thailand

**Keywords:** indoor household dust, heavy metals, health risk assessment, source identification, urban area, rural area, cancer risk

## Abstract

Indoor exposure to heavy metals poses human health risks worldwide, but study reports from Thailand are still limited, particularly in rural and urban areas. We measured the heavy metals in a hundred indoor household dust samples collected from urban and rural areas in Chiang Mai and Lamphun provinces and found a significantly higher concentration of As in rural areas and Cd in urban areas with industrial activities. The source identification of the heavy metals showed significant enrichment from traffic emissions, paint, smoking, and mixed sources with natural soil. From health risk assessment models, children were more vulnerable to noncarcinogenic risks (HI = 1.45), primarily via ingestion (HQ = 1.39). Lifetime cancer risks (LCRs) due to heavy metal exposure were found in adults (LCR = 5.31 × 10^−4^) and children (LCR = 9.05 × 10^−4^). The cancer risks from As were higher in rural areas via ingestion, while Cr and Ni were higher in urban areas via inhalation and ingestion, respectively. This study estimated that approximately 5 out of 10,000 adults and 9 out of 10,000 children among the population may develop cancer in their lifetime from exposure to indoor heavy metals in this region.

## 1. Introduction

Since the start of the COVID-19 pandemic, people around the world have been staying at home progressively more, frequently spending up to 90% of their time indoors [1,2,3]. Therefore, domestic indoor environments may represent the greatest risk factor for human exposure to indoor contaminants, especially in children, the elderly, and vulnerable people who have lived primarily indoors. According to the World Health Organization [4], in 2020, exposure to residential air pollution resulted in approximately 3.2 million deaths per year. This exposure was associated with the development of noncommunicable diseases, including stroke, ischemic heart disease, and chronic obstructive pulmonary disease (COPD). Additionally, it was determined that approximately 6% of all lung cancer deaths could be attributable to exposure to carcinogens originating from indoor household air pollution [4]. Hence, the identification, characterization, and mitigation of indoor household pollutants are important.

Indoor environments contain many pollutants, including carbon and sulfur oxides, volatile organic compounds, particulate matter, biological particles, radon, and chemicals emitted by furniture and interior decorations [5,6]. Household dust is an important indoor pollutant that contains a variety of organic and inorganic contaminants, including trace heavy metals, which can enter and harm the human body [7].

Over the past few decades, numerous studies have demonstrated that dust samples exhibit elevated concentrations of elemental species and organic pollutants [8,9,10]. The dispersion of settled dust into the atmosphere can occur through the influence of wind and various natural or human activities that have significant implications for both air quality and human health [11,12,13,14]. Furthermore, there is a correlation between the resuspension of settled dust and the presence of particulate matter indoors [15,16]. Heavy metal contamination in household dust arises from various internal and external sources of human activities. These sources include mining, vehicle emissions and transportation, fossil fuel combustion and heating methods, cooking, smoking, painting, agricultural and industrial activities, and natural sources [17,18].

The transfer of heavy metals present in dust to humans can occur through various routes of exposure, including ingestion, inhalation, and dermal absorption [19,20]. The potential health risks associated with high concentrations of toxic metals found in settled dust are a matter of concern due to their acute and chronic toxicity, especially for children and vulnerable individuals, who are more susceptible [21,22,23]. Heavy metals exhibit significant levels of persistence and biotoxicity, hence, exerting detrimental effects on several organs such as the lungs, kidneys, and other systems in the body associated with cardiovascular, cerebrovascular, skeletal, and neurological problems [24,25]. Arsenic (As) is a highly toxic substance that has been linked to the development of numerous complications in various organ systems of the body, as well as many types of cancers [26,27]. Exposure to cadmium (Cd) has been identified as a potential immunotoxicant [28], with established associations with many cancers and pronounced toxic effects on the liver and kidneys [29]. Chronic exposure and bioaccumulation of chromium (Cr) have been found to induce allergic responses, anemia, and toxicity in the male reproductive system [30]. Inhalation of manganese (Mn) has been found to have detrimental effects on the respiratory, renal, and hepatic systems, ultimately, resulting in the development of a neurological disorder known as manganism [31]. Nickel (Ni) has been associated with several health concerns, including allergies, cardiovascular and kidney problems, and lung fibrosis, as well as an increased risk of developing lung and nasal cancers [32]. Exposure to lead (Pb) has the potential to impact multiple systems inside the body, and it is especially detrimental to the neurological development of young children, as it alters the functioning of the brain and central nervous system [33]. Many studies have indicated that As, Cd, Cr, Pb, and Ni may be linked to cancer development, resulting in their classification as carcinogens [22,23,34,35,36]. Furthermore, in the body, some heavy metals exhibit a biological half-life exceeding 10–35 years, hence, presenting enduring health risks [28].

Chiang Mai and Lamphun provinces are important areas in upper Northern Thailand, which consist of urban areas, rural areas with agricultural activities, and industrial areas. These urban, industrial, and agricultural areas can be significant sources of heavy metal pollution in the form of dust from various activities. The process of urbanization has a substantial influence on the environmental quality in the area. Therefore, the primary objective of this study was to assess the concentrations of eight heavy metals in the indoor household dust in Chiang Mai and Lamphun provinces, representing urban areas in the City of Chiang Mai, industrial areas near the City of Lamphun, and agricultural areas in the rural areas of both provinces. The second objective was to identify the sources of the heavy metals in the indoor household dust of Chiang Mai and Lamphun provinces. Finally, the third objective was to evaluate a health risk assessment in terms of the carcinogenic and noncarcinogenic risks.

## 2. Materials and Methods

### 2.1. Study Area

**Figure 1:**
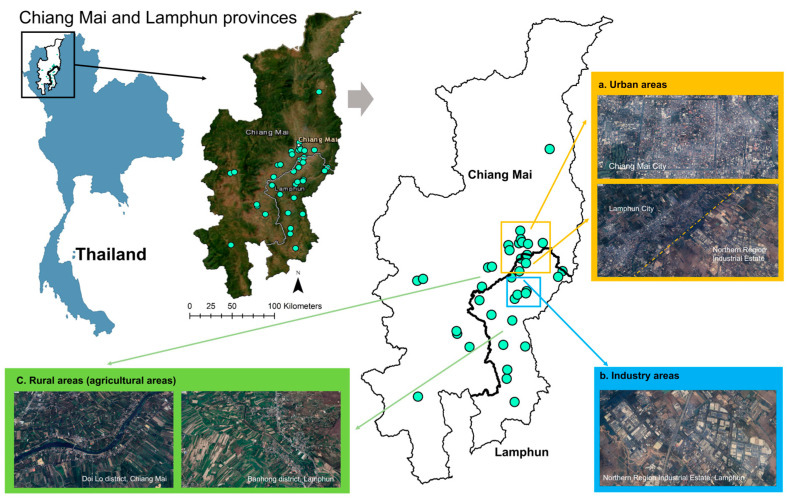
Map of the study site and the locations of collected indoor household dust.

This study was carried out within the geographical boundaries of Chiang Mai and Lamphun provinces (Figure 1). Chiang Mai and Lamphun cities are located in one of the most developed regions in Thailand, which has been undergoing rapid urbanization over the last few decades [37]. This study was conducted in urban, industrial, and rural areas with agriculture. The urban area in this study consisted of Mueang Chiang Mai district, Nai Mueang Lamphun subdistrict, and some parts of Hang Dong, San Sai, Mae Rim, and San Kamphaeng districts. The industrial area was the Industrial Estate in Lamphun province, the largest industrial area in upper Northern Thailand. These industries have primarily been engaged in the electronics, automotive components, agricultural, and manufacturing sectors. Meanwhile, the other areas of this region remain abundant in agricultural land [38]. Regardless of the forest and mountain areas, except for the urban and industrial areas, the other areas in this region were assumed to be rural areas that could be indicated as agricultural areas in this study.

### 2.2. Samples and Data Collection

Potential locations of houses were geographically mapped and house owners were informed of the study and invited to participate with their informed consent. This study was approved by the Human Experimentation Committee, Research Institute for Health Sciences (study code: Project No. 1/59, approved on: 10 May 2016). Totally, 100 settled indoor household dust samples were collected in the houses of participants. The land use types of urban, industrial, and agricultural areas of the dust-collected houses were identified within a 5 km radius during the period of dust collection. The settled dust was collected using a vacuum cleaner (HITACHI CV-SF18 220C) from surface areas of at least 1 m above the ground level of the living room and bedroom of all participants (and on the surface of furniture). Dust samples from the vacuum cleaner were dried, transferred, and filtered using a 63-micron sieve. After being sieved, approximately 0.5–2 g of dust samples was kept in a clean polyethylene Ziplock bag at −20 °C until analysis. Household characteristics and household activities were recorded during the period of dust collection.

### 2.3. Sample Analysis

To determine the heavy metal concentrations of As, Cd, Cr, Cu, Mn, Ni, Pb, and Zn in indoor settled house dust, the method of Falciani et al. was modified [39]. Briefly, 150 mg of each dust sample was digested with 5 mL of HNO_3_ and 5 mL of HCl in an ETHOS UP High-Performance Microwave Digestion System (Milestone Inc., Sorisole, Italy) using TFM microwave digestion vessels at 210 °C for 40 min. After cooling, the digested samples were diluted to 25 mL with Milli-Q water (18.2 MΩ-cm) and then filtered through a 0.2 µm nylon filter. Digestions and quality controls were analyzed for concentrations of heavy metals in triplicate with an inductively coupled plasma optical spectrometer, or ICP-OES (Agilent 5800, Agilent Scientific Technology Ltd., Santa Clara, California, USA). To prepare the calibration curve, a certified reference material, ICP Multi-Element Standard (CPA chem. Stara Zagora, Bulgaria), was used. The radiofrequency (RF) power was 1.2 kW, the nebulization flow was 0.7 L min^−1^, and the argon plasma flow was 12.0 L min^−1^. The blank experiments were conducted by repeating the steps in the sample preparation. The composition of the blank was compared with the sample solution to identify the elemental composition of the heavy metals in the dust. The calibration curves for absorbance and concentration were used to determine the concentrations of heavy metals in every sample with a straight line of r > 0.999. To ensure the method’s correctness, each heavy metal analysis was carried out in triplicate using standard reference material (SRM2584). The range of the results for the percentage recovery of standard heavy metals was 81.8% to 97.6% (Table S3).

### 2.4. Enrichment Factor (EF) Calculation

The enrichment factor (*EF*) is usually used to estimate the degree of enrichment of an element in soil and dust samples compared with its abundance in the Earth’s crust [40,41]. To determine the anthropogenic input, or the impact of human activities, in the metal values in the settled house dust, the *EF* can identify the origin of each element in the dust sample and distinguish whether that element originated from anthropogenic activity or natural sources [42] based on a reference element that is considered to be stable in its performance and not susceptible to environmental and analysis processes [43]. The *EF* was calculated using the following Equation (1).

*EF* = (*C_x_*/*C_ref_*)*_sample_*/(*C_x_*/*C_ref_*)*_background_* (1)

where *C_x_* is the examined metal concentration, and *C_ref_* is the reference metal concentration for normalization. In this study, Mn was applied as the reference metal [14]. The background values of the chemical elements in the continental crust followed Taylor’s report [44].

### 2.5. Health Risk Assessment

The exposure dose to the heavy metals in the house dust was estimated via three routes of exposure [45]. The model developed by the US Environmental Protection Agency [46] was used to calculate the exposure to metals in settled house dust. The average daily doses (*ADDs*, mg kg^−1^ day^−1^) of heavy metals in household dust via ingestion, dermal contact, and inhalation as exposure pathways were calculated separately using Equations (2)–(4) for noncarcinogenic risk and (5)–(7) for carcinogenic risk [14,47]. The average daily doses (*ADDs*, mg kg^−1^ day^−1^) for noncarcinogenic risk were calculated as follows:

(2) $$ADDingest=C\times \frac{IngR\times ExF\times ED}{BW\times AT}\times CF$$

(3) $$ADDinhal=C\times \left[\frac{\;InhR\times ExF\times ET\times ED}{PEF\times BW\times AT}\right]$$

(4) $$ADDdermal=C\times \left[\frac{\;SA\times SL\times ABS\times ExF\times ED}{BW\times AT}\right]\times CF$$

The average daily doses (*ADDs*, mg kg^−1^ day^−1^) for carcinogenic risk were calculated as follows:

(5) $$ADDingest_{carcinogenic}=C\times \left[\frac{\;IR\times ExF\;}{AT}\right]\times CF$$

(6) $$ADDinhal_{carcinogenic}=C\times \left[\frac{\;ExF\times ET\times ED}{PEF\times 24\times AT}\right]\times 1000$$

(7) $$ADDdermal_{carcinogenic}=\;C\times \left[\frac{\;ABS\times ExF\times DFS}{AT}\right]\times CF$$

where *C* is the measured concentration of heavy metals in household dust (mg kg^−1^), and *IngR* and *InhR* are the ingestion and inhalation rates, respectively; *ExF* is the exposure frequency (day year^−1^); *ED* is the exposure duration, represented by years of stay in the house (years), obtained from the questionnaire; *BW* is the body weight (kg); *AT* is the average time stay in the house per day; *PEF* is the particle emission factor; *SL* is the skin adherence factor; *SA* is the exposed skin area; and *ABS* is the dermal absorption factor. The values of the parameters are listed in Table S1.

Subsequently, the hazard index and cancer risk methods were used to assess the health risks of heavy metal exposure to household dust. The calculated doses of each metal for the three exposure pathways were compared to their corresponding reference dose (*RfD*) (mg kg^−1^ day^−1^) to yield a hazard quotient (*HQ*), as shown in Equation (8), and then summed to obtain the total noncarcinogenic risk of all pathways using the hazard index (*HI*), as shown in Equation (9) [46]. For noncarcinogenic risk, if the *HQ* or *HI* < 1, this indicates that there is no significant risk effect. In contrast, if the *HQ* or *HI* ≥ 1, there is possibly a noncarcinogenic effect, which tends to increase in effect when the *HQ* or *HI* increases [46].

(8) $$HQ=\frac{ADD\;}{RfD}$$

(9) $$HI=\sum HQ_{Ingest}+HQ_{Inhal}+HQ_{Dermal}$$

While the carcinogenic risk, or cancer risk (*CR*), estimates the carcinogenic effects over the lifetime, the dose was multiplied by the corresponding cancer slope factor (*SF*) [(mg kg^−1^ day^−1^)]^−1^. Total cancer risks, or lifetime cancer risk (*LCR*), were assessed by summing the cancer risk of each exposure route [13,46], which is defined as follows:

(10) CR=ADD×SF

(11) $$LCR=\sum CR_{Ingest}+CR_{Inhal}+CR_{Dermal}$$

For the carcinogenic risk, if the *CR* or *LCR* is in a range between 1 × 10^−6^ and 1 × 10^−4^, this can indicate an acceptable or tolerable risk; if higher than 1 × 10^−4^, this suggests that, at least, 1 in 10,000 people may develop any cancer from lifetime exposure [14]. The reference values for the noncarcinogenic and carcinogenic risk assessment are shown in Table S2.

### 2.6. Source Apportionment Model

In environmental research, positive matrix factorization (PMF) is typically utilized to identify and allocate pollution sources according to the composition of the pollutants measured at a certain location. It is frequently used to evaluate complex combinations of contaminants and to determine how various sources contribute to the total contamination. To evaluate the source apportionment of heavy metals in indoor settled household dust, PMF was employed. According to the US EPA, the PMF 5.0 user manual (version 5.0.14) software was used. Source identification was established according to representative elements of base run factors that were selected by sample concentration. To determine the source contributions and component profiles of the pollutant sources under non-negative limitations, the concentration of the elements and uncertainty values were employed in PMF [48], as per the following Equation (12):

(12) $$x_{ij}=\overset{p}{\underset{k=1}{\sum }}\left(g_{ik}f_{kj}+e_{ij}\right)$$

where *X_ij_* is the concentration of a species, *g_ik_* is the factor contribution, *f_kj_* is the factor profile, *e_ij_* is the residual matrix, *p* is the factor number, *i* is the sample number, and *j* is the species of element.

To produce the ideal matrices of G and F by repeatedly breaking down the heavy metal concentration matrix X, PMF minimizes the objective function *Q*, as per the following Equation (13):

(13) $$Q=\overset{n}{\underset{i=1}{\sum }}\overset{m}{\underset{j=1}{\sum }}{\left[\frac{e_{ij}}{u_{ij}}\right]}^{2}$$

The uncertainty is calculated using a fixed fraction of the method detection limit (*MDL*) according to the EPA PMF 5.0 user guide, as shown in Equations (14) and (15).

When concentration less than the *MDL*:

(14) $$u_{ij}=\frac{5}{6}\times MDL$$

When concentration more than *MDL*:

(15) $$u_{ij}=\sqrt{error\;fraction\times c+MDL}$$

The numbers of the F (factor profile) and G (factor contribution) matrices are examined with FPEAK and classified as “rotational ambiguity”, which can be used to calculate the minimum Q value. PMF could be performed on five FPEAK runs in this dataset. Every variable had a strong signal-to-noise ratio and no missing values or outliers in the samples. Thirty runs of the model yielded five possible source assumptions. Model residual analysis and model diagnostics were utilized to ascertain the five possible components. The mapping of the bootstrap factors to the base factors was over 80%, indicating that the bootstrap uncertainties could be interpreted and appropriated to the number of factors.

### 2.7. Statistical Analysis

According to the normality distribution test, most of the heavy metal levels showed a non-normal distribution (*p* < 0.001, except for Mn). Therefore, nonparametric tests, including the Kruskal–Wallis and Mann–Whitney tests, were used to compare the median values of the datasets. A *p*-value of <0.05 shows significant differences between the median of the compared groups.

## 3. Results and Discussion

### 3.1. Heavy Metal Concentrations in Indoor Household Dust

**Figure 2:**
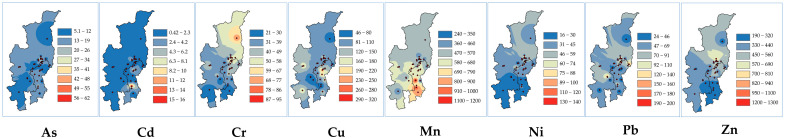
Distribution map of heavy metal concentrations (mg kg^−1^) in indoor household dust using the inverse distance weighting (IDW) interpolation method.

The median and IQR concentrations (mg kg^−1^) of eight heavy metals in household dust are shown in Table 1. We found that Mn (542.7, 270.3 mg kg^−1^) exhibited the highest concentration in indoor household dust followed by Zn (352.7, 246.9 mg kg^−1^), >Cu (82.5, 56.3 mg kg^−1^), >Pb (44.8, 27.7 mg kg^−1^), >Cr (32.4, 18.3 mg kg^−1^), >Ni (28.9, 17.9 mg kg^−1^), >As (10.3, 6.1 mg kg^−1^), and Cd (0.9, 1.5 mg kg^−1^), which was the lowest concentration. The concentration ranges of As, Cd, Cr, Cu, Mn, Ni, Pb, and Zn in indoor household dust in the present study were from 4.2 to 62.1 mg kg^−1^, 0.2 to 20.2 mg kg^−1^, 17.2 to 148.0 mg kg^−1^, 25.1 to 401.5 mg kg^−1^, 204.8 to 1318.1 mg kg^−1^, 11.2 to 146.2 mg kg^−1^, 18.0 to 426.4 mg kg^−1^, and 123.8 to 1527.2 mg kg^−1^, respectively. The distribution map of the heavy metal concentrations in indoor household dust in Chiang Mai and Lamphun provinces is shown in Figure 2.

The concentrations of heavy metals in this study were within the range of previous studies worldwide (Table 2). However, in contrast to other studies, only Mn in this study was different, changing the order and showing a higher concentration than Zn in this region. Regardless of the highest value of Mn, the orders of the heavy metal concentrations in this region are likely similar to those found in China, the UK, a meta-analysis of 35 countries [17], and other countries, with slight changes in the order for Cu, Pb, Cr, and Ni (Table 2). The higher concentration of Mn than Zn in this study may be due to the origin of the household dust from contamination by outdoor air and soil resuspension, which is highly abundant in Mn. While Zn-rich content was related to industrial emissions and urban areas [14,17], there was also found a higher concentration of Zn in urban areas than in rural areas with a borderline significant difference in the present study (Table 3). Additionally, in the case of this study, the geological background might be a crucial factor in the metal content. Some studies revealed that detached homes had higher levels of Mn [17,49], and most houses in this study were detached single houses (data are not shown). Moreover, most houses in Northern Thailand are open-air houses, which allows natural ventilation throughout by keeping the doors and windows open [6]. Soil dust that contains abundant Mn can easily enter houses. In addition, agricultural chemicals are a potential source of Mn [50], and most people in this region are engaged in agricultural work that easily transfers contaminated soil and agricultural chemicals onto the body and into homes.

Regarding the differences in urbanization, the concentrations of heavy metals in urban areas, industrial areas, and agricultural areas, such as rural areas, were investigated. The comparison of the heavy metal concentrations (mg kg^−1^) in indoor household dust in the different areas is shown in Table 3. On the basis of the results, a significantly higher concentration of As was found in rural areas than urban areas, and a borderline significantly higher concentration of Zn was found in urban areas than rural areas. Meanwhile, the concentrations of Cd, Cr, Cu, Mn, Ni, and Pb showed no significant differences between urban and rural areas. It was previously reported that the As level decreases with an increase in the distance from the city center [17] and is related to pesticide use in agricultural lands [35,51]. Moreover, the higher concentration of As in rural areas could be linked to the contamination of outdoor soil [40], while Zn was mostly linked to activities in urban areas, such as vehicle transportation [14] and industrial activities [17].

In the industrial area, there was a significantly higher concentration of only Cd in the indoor household dust of the houses located near the industrial area than in the houses far from the industrial area. While for the other metals, there were found no significant differences. It has previously been reported that emissions of Cd mainly originate from various industrial activities [18,52]. In addition, electronic waste recycling in electronic industries also played a significant part in Cd contamination [52]. The differences in the heavy metal concentrations in the different areas suggests that anthropogenic activities can influence a variety of heavy metals.

**Table 2:** A comparison of heavy metals in indoor dust with previous studies.

Countries (*n*)	Heavy Metal Concentrations in Indoor Dust (mg kg^−1^)								Ref.
	As	Cd	Cr	Cu	Mn	Ni	Pb	Zn	
35 Countries (*n* = 2265) ^a^	25.3		128	264	333	77.6	224	1470	[17]
35 Countries (*n* = 2265) ^b^	13.3	0.76	86	176	257	39	94	1110	[17]
Australia (*n* = 1310) ^a^	31.9		105	232	336	49.4	305	1680	[17]
China (*n* = 111) ^a^	17.3		134	242	245	70.6	161	1190	[17]
Ghana (*n* = 54) ^a^	6.2		43.4	74.9	185	48	163	252	[17]
UK (*n* = 148) ^a^	6.9		93.6	136	269	35.8	131	532	[17]
USA (*n* = 345) ^a^	20.5		207	549	385	165.4	93.6	1785	[17]
Turkey (*n* = 85) ^b^	4.41	0.35	23.8	65.7	65.9	32.3	27.5	263	[53]
Australia (*n* = 224) ^a^	20.2		99.8	298	247	56.7	364	2437	[47]
Across China (*n* = 3392) ^b^	15.6	2.73	85.9	136.2		40.7	161.5	602.7	[18]
Canada (*n* = 125) ^a^	13	11	92	1900	250	60	4500	14,000	[54]
UK (*n* = 32) ^c^		1.2		301	524	53.1	150	622	[55]
Japan (*n* = 100) ^c^		1.02	67.8	304	226	59.6	57.9	920	[56]
Sukhothai, Thailand (*n* = 16) ^a^		9		3			226	1051	[57]
Ubon Ratchathani, Thailand (*n* = 56) ^a^			0.99			0.92	3.00		[58]
This study (*n* = 100) ^a^	12.5	2.2	38.5	107.3	577.6	34.9	62.1	408.7	
This study (*n* = 100) ^b^	10.3	0.9	32.4	82.5	542.7	28.9	44.8	352.7	

^a^ Mean. ^b^ Median. ^c^ Geomean.

**Table 3:** A comparison of the heavy metal concentrations (mg kg^−1^) in indoor household dust in urbanized areas, agricultural areas, and industrial areas in Chiang Mai and Lamphun provinces.

Areas	Median, IQR Concentration, (mg kg^−1^)							
	As	Cd	Cr	Cu	Mn	Ni	Pb	Zn
Urbanized								
Rural (agriculture) (79)	10.97, 5.89	0.89, 1.31	30.97, 18.00	79.18, 57.25	577.41, 284.14	27.37, 18.93	45.25, 29.18	332.33, 193.00
Urban (21)	8.80, 4.14	1.08, 1.53	41.89, 15.12	84.13, 57.58	461.83, 288.92	30.94, 17.36	44.16, 41.19	420.46, 234.74
*p*-Value	0.042	0.308	0.137	0.557	0.102	0.245	0.632	0.053
Industrial area nearby								
Yes (21)	10.97, 6.20	1.37, 9.49	34.61, 16.33	81.83, 34.49	519.73, 205.02	29.86, 24.55	49.29, 31.13	384.10, 199.74
No (74)	10.25, 6.50	0.83, 1.18	31.41, 19.91	84.16, 63.33	573.72, 281.19	27.92, 16.89	42.18, 30.28	344.83, 214.65
*p*-Value	0.784	0.028	0.654	0.438	0.510	0.319	0.247	0.654

### 3.2. Source Identification of Heavy Metal in Indoor Household Dust

Source identification was conducted using source modeling of the enrichment factor (EF) and positive matrix factorization (PMF). The *EF* values can indicate the sources of influence, whether anthropogenic activities or natural sources, on dust and soil concentrations when compared to Mn as the crush metal [42].

The mean value of the *EF* for each heavy metal in the indoor household dust is shown in Table 4. The *EF* values of the heavy metals in the indoor household dust are ranked in the order of Cd (18.2) followed by As (11.1) > Zn (10.8) > Pb (8.0) > Cu (3.8) > Ni (0.8) > Cr (0.7). The *EF* can indicate the natural conditions when the value is less than 2 and suggest an anthropogenic influence when it is greater than 2 [59]. On the basis of the results, the mean *EF* values indicated an extremely high enrichment of Cd, As, and Zn, which were higher than 10, suggesting a significant enrichment level derived from anthropogenic activity [59,60]. The *EF* values of Pb and Cu were between 2 and 10, indicating that these metals were lightly enriched and mainly originated from human activities. The *EFs* of Ni and Cr were lower than 1, therefore, indicating that they certainly originated from natural sources and were not affected by human activities. Regarding anthropogenic activities and heavy metals, As, Cd, and Zn have been reported as potential metals from agricultural chemical sources [50,51] and industrial activities [18,52]. Moreover, As, Pb, and Zn are reportedly related to traffic emissions and motor vehicles [14,17,61]. Paint is associated with increased As, Cu, Pb, and Zn in household dust [14,18,62,63]. Regarding natural sources, Cr and Ni, in this study, were identified as contaminants from a natural origin, like natural soil. Soil is the dominant contributor of Ni and Cr to household dust [40,56]. Nevertheless, some heavy metals can originate from both natural and anthropogenic sources [64]. Moreover, the simultaneous accumulation of metals from soil and anthropogenic sources can enhance the enrichment of heavy metals in household dust, which may lead to increased *EF* values.

**Figure 3:**
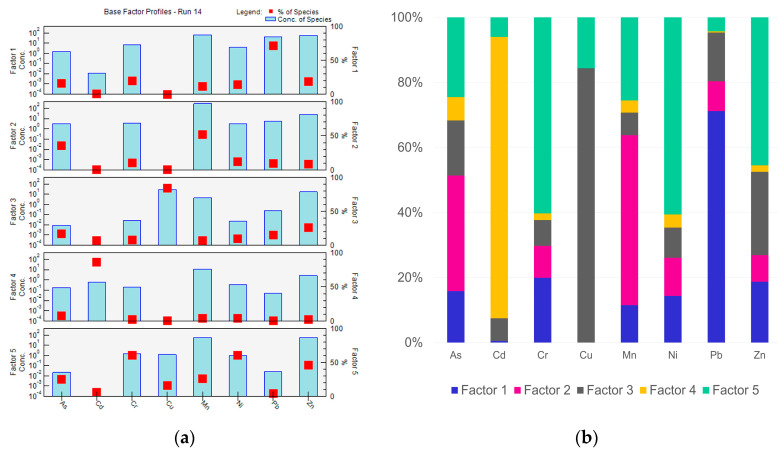
Factor profiles and factor fingerprints plot of 8 heavy metals in the indoor household dust in this study: (**a**) factor fingerprints plot; (**b**) factor profiles.

For further source identification, the PMF model was employed. The results show the heavy metals in the house dust in this study mainly came from five sources. The factor fingerprints plot (Figure 3) illustrates the distribution of the metal species by the various sources and provides the percentage contribution of the metal species according to the various sources. Factor profiles are shown in Figure 3. Factor 1 explains 19.01%, factor 2 explains 15.83%, factor 3 explains 21.64%, factor 4 explains 13.24%, and factor 5 explains 30.28% of all data for the heavy metals in the indoor household dust.

Factor 1 was dominated by Pb, which was attributed to traffic emissions and the painting of houses. It has been reported that Pb is primarily related to leaded gasoline usage, and it is associated with house dust in homes with a high traffic density nearby [17,40,61,65,66]. Paint and coating materials were also related to the higher concentration of Pb in house dust [14,18,66,67].

As and Mn were high in concentration, as explained by factor 2, representing natural soil and agriculture. Soil and the Earth’s crust are the most important sources of these metals [17,40,64].

Factor 3, represented by Cu, was caused by paint pigment in houses. The As, Cu, Pb, and Zn in the household dust were also associated with colored paints for houses, building materials, and stainless steel used in household furniture [14,17,62]. Wall paint and coating materials may lead to the accumulation of Cu in house dust, especially green paint [63].

Factor 4 was dominated by Cd, which is attributed to cigarette smoking. Several studies have reported tobacco smoking to be an important factor of elements enriched in house dust, particularly Cd, Ni, Pb, and Zn [14,68], as cigarettes contain these elements [69,70]. However, Cd is also used in color pigments for paints and coating materials [14,18], and they originate from agricultural chemicals, traffic emissions, and wildfires [14,17,18,50]. Probably, the sources of Cd are not only contamination from tobacco smoke but also from other mixed sources.

Factor 5 may be identified as multiple sources or a complex mixture of outdoor sources including natural sources of Cr and Ni and anthropogenic sources of Zn. It has been reported that Cr and Ni can originate from soil [17,64]. Moreover, Zn in household dust has been related to outdoor agriculture [50], motor vehicles [14], and paint [18]. Additionally, the very high enrichment of Zn content might not be of natural origin and must come from either industrial emissions [17] or from the geological background of this area.

### 3.3. Health Risk Assessment

The human health risks of carcinogenic and noncarcinogenic risks were calculated as a result of exposure to heavy metals in indoor household dust via inhalation, ingestion, and dermal contact routes. The calculated carcinogenic and noncarcinogenic risks of the heavy metals are shown in Table 5 and Table 6, respectively. For the noncarcinogenic risk, the hazard quotient (*HQ*) exhibits the main risk of the heavy metals in the household dust from ingestion, followed by the dermal contact and inhalation exposure pathways. Except for only Cu in adults and Mn in children, the highest exposure pathway was ingestion followed by inhalation and dermal contact. The hazard index (*HI*) or overall health risk attributable to exposure to heavy metals in household dust via all tree pathways in adults and children were in the same order as As > Mn > Pb > Cr > Cd > Cu > Ni > Zn. On the basis of these results, in rural and urban areas, the overall *HI* values of all heavy metals in adults were lower than 1, suggesting that the heavy metals in household dust were within tolerable limits or had no effect on noncarcinogenic risk in this study. However, the *HI* in children exhibited noncarcinogenic risk primarily through ingestion. Suggesting that there was a possibility of noncarcinogenic effects in children in this area from heavy metal exposure. The finding of noncancer risk only in children may be due to children exhibiting a greater vulnerability to indoor heavy metal contamination than adults. This susceptibility is attributed to their lower body weight and higher dust ingestion rates resulting from increased physical activity [17,18]. Consequently, children face a higher health risk from indoor heavy metals than adults.

For the carcinogenic risks, As, Cd, Cr, Ni, and Pb are known as carcinogenic metals [35]. Hence, the carcinogenic risks of these metals in the indoor household dust were calculated. The overall total risk or lifetime cancer risk (*LCR*) of carcinogenic heavy metal exposure was highest in the inhalation pathway, followed by ingestion and dermal contact pathways, respectively. In adults, the *LCR* values via the three pathways decreased in the following order: Cr > As > Cd > Ni > Pb. From the calculation, Cr exhibited a carcinogenic risk through inhalation in rural and urban areas. The total carcinogenic risks of the eight metals were found in the ingestion and inhalation pathways, while there was no cancer risk in the dermal contact pathway. On the basis of these results, the inhalation exposure pathway showed a higher cancer risk than the ingestion pathway. The higher inhalation risk may be due to the inhalation cancer risk from Cr exposure (3.89 × 10^−4^) that was more significant than 1 × 10^−4^, an unacceptable level for carcinogenic risk, while Cd, As, Ni, and Pb were between 1 × 10^−4^ and 1 × 10^−6^, which are regarded as acceptable or tolerable risks. Hence, this study indicates that inhaled Cr in indoor household dust poses a cancer risk and suggests the inhalation of Cr in household dust is the dominant exposure pathway to an attributable risk to cancer in adults in this region. An unacceptable level of Cr in indoor dust was found in most cities in China, Turkey, Australia, and New Caledonia and was most dominant through the inhalation pathway [17,18,47,71]. In epidemiologic studies, Cr was reported to be correlated with lung cancer, usually in chromate-related occupational workers [72,73,74]. Chromium (VI) compounds are also classified as group 1 carcinogenic to humans, and there is sufficient evidence causing lung cancer [35]. Even though there are two common forms of Cr, including Cr(VI) and Cr(III), Cr(VI) was the primary toxicity of Cr exposure. In addition, the proportions of Cr(VI) to Cr(III) can vary widely, ranging from 0.5 to 2.5 [75], and are influenced by factors such as pH, chemical reactions, biological processes, and environmental conditions [76]. Hence, the parameters for the health risk assessment of Cr in household dust in this study were from Cr(VI) due to the greater toxicity that might be higher than the actual risk. In addition, cancer risk through inhalation was higher in urban areas than rural areas, while the carcinogenic risk via ingestion was higher in rural areas than urban areas. This may be due to the higher concentration of Cr in urban areas that positively correlated to the city area [17,77]. Consequently, this study suggests that 5.3 in 10,000 adults (4 from inhalation and 1.3 from ingestion exposure) may develop any cancer from lifetime exposure to these heavy metals in indoor household dust. It is important to note that the carcinogenic risk from inhalation of Cr may be the crucial factor for the high incidence of respiratory diseases, such as COPD, asthma, and lung cancer, in this region of Northern Thailand [78]. Moreover, lung cancer is reported to have a high incidence in Northern Thailand and continues to have significantly higher incidence and mortality annually compared to the other parts [78,79,80]. Interestingly, heavy metals such as Cr in household dust might be another cause of respiratory diseases development in this region, particularly lung cancer.

In children, the overall *LCR* value of the three pathways decreased in the following order: Cr > As > Ni > Pb > Cd. On the basis of the results, As (*LCR* = 3.21 × 10^−4^), Cr (*LCR* = 4.29 × 10^−4^), and Ni (*CR_ing_* = 1.03 × 10^−4^) exhibited carcinogenic risks in children in this study. Through ingestion, As and Cd showed cancer risk in urban and rural areas, while Ni revealed a cancer risk only in urban areas. Likewise, Cr via inhalation was found only to show a cancer risk in urban areas. This might be due to urban areas having higher levels of these metals [17], as well as the correlation with vehicle emissions in the city [14,61,81]. The carcinogenic risks in children were different from those in adults, and it was found that the dominant risk was greater through ingestion than in the inhalation pathway. This is due to children’s behaviors, which lead to higher dust ingestion rates, and lower body weights than adults [17,18]. From the overall *LCR*, this study suggests that 9 in 10,000 children (8 from ingestion and 1 from inhalation exposure) may develop any cancer from lifetime exposure to these heavy metals in indoor household dust.

## 4. Conclusions

This study investigated indoor pollutants, with a particular focus on heavy metal contamination in household dust as a significant health risk factor. The study was conducted in Chiang Mai and Lamphun provinces in Northern Thailand, encompassing urban, industrial, and agricultural areas and contributions to heavy metal pollution through various activities. The research found elevated concentrations of heavy metals in indoor household dust, with Mn showing the highest concentration, followed by Zn > Cu > Pb > Cr > Ni > As > Cd. Using the *EFs* and PMF, we identified the sources of the heavy metals in the indoor dust, which indicated that Cd, As, and Zn were significantly enriched by anthropogenic activities, while Pb and Cu showed moderate enrichment. Factors such as traffic emissions, painting, agriculture, and industrial activities were identified as key contributors to heavy metal contamination in the household dust. Health risk assessments were conducted for both the carcinogenic and noncarcinogenic risks associated with the heavy metals’ exposure. The results suggest that the noncarcinogenic risks, primarily from ingestion, could pose potential health effects in children. For adults, the noncarcinogenic risks were generally within tolerable limits. Regarding the carcinogenic risks, Cr exposure through inhalation was identified as the most significant risk for adults. Urban areas exhibited higher inhalation risks due to elevated Cr concentrations and associated vehicle emissions. In children, the carcinogenic risks were observed primarily through ingestion, with significant risks associated with As, Cr, and Ni. This study demonstrates the health risks from heavy metals exposure via indoor household dust in both children and adults from Chiang Mai and Lamphun provinces, Northern Thailand. These findings support the previous scientific knowledge that heavy metals in indoor house dust can be a risk to human health, particularly cancer risks. To the best of our knowledge, our study is the first of its kind with a sample size of hundred and eight heavy metals from Thailand. However, the exposure assessment among both adults and children in high-risk areas is worth being further explored.

## Figures and Tables

**Table 1:** The concentrations of heavy metals (mg kg^−1^) in indoor household dust in this study.

Heavy Metals	Concentration (mg kg^−1^)					
	Mean	SD	Median	IQR	Min.	Max.
As	12.5	8.8	10.3	6.1	4.2	62.1
Cd	2.2	3.9	0.9	1.5	0.2	20.2
Cr	38.5	20.1	32.4	18.3	17.2	148.0
Cu	107.3	78.2	82.5	56.3	25.1	401.5
Mn	577.6	222.5	542.7	270.3	204.8	1318.1
Ni	34.9	23.6	28.9	17.9	11.2	146.2
Pb	62.1	57.8	44.8	27.7	18.0	426.4
Zn	408.7	246.9	352.7	210.1	123.8	1527.2

**Table 4:** The enrichment factor of heavy metals in indoor household dust.

Heavy Metals	*n*		Enrichment Factor				
		Mean	SD	Median	IQR	Min.	Max.
As	100	11.13	5.66	10.13	4.93	3.82	47.88
Cd	96	18.18	27.69	9.43	13.41	1.47	141.17
Cr	100	0.70	0.48	0.61	0.39	0.21	4.15
Cu	97	3.76	3.24	2.87	2.14	0.72	19.77
Ni	99	0.84	0.61	0.65	0.50	0.18	3.60
Pb	100	7.99	5.14	6.48	5.03	2.41	27.99
Zn	99	10.84	7.68	9.21	8.77	1.85	51.82

**Table 5:** Noncarcinogenic risk or hazard quotient (*HQ*) of 8 heavy metals in household dust in Chiang Mai and Lamphun provinces.

Heavy Metal	Noncarcinogenic Risk							
	Adult				Child			
	HQ_ing_	HQ_inh_	HQ_dermal_	HI	HQ_ing_	HQ_inh_	HQ_dermal_	HI
As	6.20 × 10^−2^	1.49 × 10^−4^	1.81 × 10^−2^	8.02 × 10^−2^	5.21 × 10^−1^	2.91 × 10^−4^	1.81 × 10^−2^	5.39 × 10^−1^
Urban	5.79 × 10^−2^	1.39 × 10^−4^	1.69 × 10^−2^	7.49 × 10^−2^	4.86 × 10^−1^	2.72 × 10^−4^	1.69 × 10^−2^	5.03 × 10^−1^
Rural	6.31 × 10^−2^	1.51 × 10^−4^	1.84 × 10^−2^	8.17 × 10^−2^	5.30 × 10^−1^	2.96 × 10^−4^	1.84 × 10^−2^	5.49 × 10^−1^
Cd	1.09 × 10^−2^	2.29 × 10^−4^	1.74 × 10^−3^	1.28 × 10^−2^	9.14 × 10^−2^	4.48 × 10^−4^	8.68 × 10^−3^	1.01 × 10^−1^
Urban	3.23 × 10^−3^	6.79 × 10^−5^	5.15 × 10^−4^	3.81 × 10^−3^	2.71 × 10^−2^	1.33 × 10^−4^	2.57 × 10^−3^	2.98 × 10^−2^
Rural	1.29 × 10^−2^	2.72 × 10^−4^	2.06 × 10^−3^	1.53 × 10^−2^	1.09 × 10^−1^	5.32 × 10^−4^	1.03 × 10^−2^	1.19 × 10^−1^
Cr	1.94 × 10^−2^	6.99 × 10^−5^	3.88 × 10^−3^	2.34 × 10^−2^	1.63 × 10^−1^	1.37 × 10^−4^	1.94 × 10^−2^	1.83 × 10^−1^
Urban	2.09 × 10^−2^	7.50 × 10^−5^	4.16 × 10^−3^	2.51 × 10^−2^	1.75 × 10^−1^	1.47 × 10^−4^	2.08 × 10^−2^	1.96 × 10^−1^
Rural	1.90 × 10^−2^	6.85 × 10^−5^	3.80 × 10^−3^	2.29 × 10^−2^	1.60 × 10^−1^	1.34 × 10^−4^	1.90 × 10^−2^	1.79 × 10^−1^
Cu	4.99 × 10^−3^	3.47 × 10^−5^	6.64 × 10^−5^	5.09 × 10^−3^	4.19 × 10^−2^	6.79 × 10^−5^	3.32 × 10^−4^	4.23 × 10^−2^
Urban	3.88 × 10^−3^	2.70 × 10^−5^	5.16 × 10^−5^	3.96 × 10^−3^	3.26 × 10^−2^	5.28 × 10^−5^	2.58 × 10^−4^	3.29 × 10^−2^
Rural	5.29 × 10^−3^	3.68 × 10^−5^	7.04 × 10^−5^	5.40 × 10^−3^	4.44 × 10^−2^	7.20 × 10^−5^	3.51 × 10^−4^	4.49 × 10^−2^
Mn	3.64 × 10^−2^	2.10 × 10^−3^	1.90 × 10^−3^	4.04 × 10^−2^	3.06 × 10^−1^	4.10 × 10^−3^	9.47 × 10^−3^	3.20 × 10^−1^
Urban	3.19 × 10^−2^	1.83 × 10^−3^	1.66 × 10^−3^	3.53 × 10^−2^	2.68 × 10^−1^	3.59 × 10^−3^	8.28 × 10^−3^	2.79 × 10^−1^
Rural	3.77 × 10^−2^	2.17 × 10^−3^	1.96 × 10^−3^	4.18 × 10^−2^	3.16 × 10^−1^	4.24 × 10^−3^	9.79 × 10^−3^	3.30 × 10^−1^
Ni	2.69 × 10^−3^	3.13 × 10^−7^	3.97 × 10^−5^	2.73 × 10^−3^	2.26 × 10^−2^	6.12 × 10^−7^	1.98 × 10^−4^	2.28 × 10^−2^
Urban	2.94 × 10^−3^	3.42 × 10^−7^	4.35 × 10^−5^	2.98 × 10^−3^	2.47 × 10^−2^	6.70 × 10^−7^	2.17 × 10^−4^	2.49 × 10^−2^
Rural	2.62 × 10^−3^	3.05 × 10^−7^	3.87 × 10^−5^	2.66 × 10^−3^	2.20 × 10^−2^	5.97 × 10^−7^	1.93 × 10^−4^	2.22 × 10^−2^
Pb	2.68 × 10^−2^	3.19 × 10^−6^	7.12 × 10^−4^	2.75 × 10^−2^	2.25 × 10^−1^	6.25 × 10^−6^	3.56 × 10^−3^	2.28 × 10^−1^
Urban	2.81 × 10^−2^	3.34 × 10^−6^	7.46 × 10^−4^	2.88 × 10^−2^	2.36 × 10^−1^	6.55 × 10^−6^	3.73 × 10^−3^	2.39 × 10^−1^
Rural	2.64 × 10^−2^	3.15 × 10^−6^	7.03 × 10^−4^	2.71 × 10^−2^	2.22 × 10^−1^	6.17 × 10^−6^	3.51 × 10^−3^	2.25 × 10^−1^
Zn	2.11 × 10^−3^	2.53 × 10^−7^	4.22 × 10^−5^	2.16 × 10^−3^	1.78 × 10^−2^	4.96 × 10^−7^	2.11 × 10^−4^	1.80 × 10^−2^
Urban	2.66 × 10^−3^	3.18 × 10^−7^	5.30 × 10^−5^	2.71 × 10^−3^	2.23 × 10^−2^	6.24 × 10^−7^	2.65 × 10^−4^	2.26 × 10^−2^
Rural	1.97 × 10^−3^	2.36 × 10^−7^	3.93 × 10^−5^	2.01 × 10^−3^	1.65 × 10^−2^	4.62 × 10^−7^	1.96 × 10^−4^	1.67 × 10^−2^
Total	1.65 × 10^−1^	2.58 × 10^−3^	2.65 × 10^−2^	1.94 × 10^−1^	1.39	5.06 × 10^−3^	5.99 × 10^−2^	1.45
Urban	1.51 × 10^−1^	2.15 × 10^−3^	2.41 × 10^−2^	1.78 × 10^−1^	1.27	4.20 × 10^−3^	5.30 × 10^−2^	1.33
Rural	1.69 × 10^−1^	2.70 × 10^−3^	2.71 × 10^−2^	1.99 × 10^−1^	1.42	5.28 × 10^−3^	6.17 × 10^−2^	1.49

**Table 6:** Carcinogenic risk (*CR*) and lifetime cancer risk (*LCR*) of 8 heavy metals in household dust in Chiang Mai and Lamphun provinces.

Heavy Metals	Carcinogenic Risk (CR)							
	Adult				Child			
	CR_ing_	CR_inh_	CR_dermal_	LCR	CR_ing_	CR_inh_	CR_dermal_	LCR
As	5.02 × 10^−5^	1.27 × 10^−8^	2.73 × 10^−6^	5.30 × 10^−5^	3.18 × 10^−4^	3.18 × 10^−9^	2.73 × 10^−6^	3.21 × 10^−4^
Urban	4.69 × 10^−5^	1.19 × 10^−8^	2.55 × 10^−6^	4.94 × 10^−5^	2.97 × 10^−4^	2.96 × 10^−9^	2.55 × 10^−6^	2.99 × 10^−4^
Rural	5.11 × 10^−5^	1.29 × 10^−8^	2.78 × 10^−6^	5.39 × 10^−5^	3.24 × 10^−4^	3.23 × 10^−9^	2.78 × 10^−6^	3.26 × 10^−4^
Cd	7.44 × 10^−6^	1.22 × 10^−5^	5.39 × 10^−7^	2.02 × 10^−5^	4.71 × 10^−5^	3.05 × 10^−6^	5.39 × 10^−7^	5.07 × 10^−5^
Urban	2.21 × 10^−6^	3.61 × 10^−6^	1.60 × 10^−7^	5.98 × 10^−6^	1.40 × 10^−5^	9.04 × 10^−7^	1.60 × 10^−7^	1.50 × 10^−5^
Rural	8.84 × 10^−6^	1.45 × 10^−5^	6.41 × 10^−7^	2.39 × 10^−5^	5.60 × 10^−5^	3.62 × 10^−6^	6.41 × 10^−7^	6.02 × 10^−5^
Cr	5.25 × 10^−5^	3.89 × 10^−4^	9.50 × 10^−8^	4.41 × 10^−4^	3.32 × 10^−4^	9.72 × 10^−5^	9.50 × 10^−8^	4.29 × 10^−4^
Urban	5.63 × 10^−5^	4.18 × 10^−4^	1.02 × 10^−7^	4.74 × 10^−4^	3.57 × 10^−4^	1.04 × 10^−4^	1.02 × 10^−7^	4.61 × 10^−4^
Rural	5.14 × 10^−5^	3.81 × 10^−4^	9.32 × 10^−8^	4.33 × 10^−4^	3.26 × 10^−4^	9.53 × 10^−5^	9.32 × 10^−8^	4.21 × 10^−4^
Ni	1.49 × 10^−5^	2.22 × 10^−9^	1.59 × 10^−7^	1.51 × 10^−5^	9.43 × 10^−5^	5.55 × 10^−10^	1.59 × 10^−7^	9.45 × 10^−5^
Urban	1.63 × 10^−5^	2.43 × 10^−9^	1.75 × 10^−7^	1.65 × 10^−5^	1.03 × 10^−4^	6.07 × 10^−10^	1.75 × 10^−7^	1.03 × 10^−4^
Rural	1.45 × 10^−5^	2.16 × 10^−9^	1.55 × 10^−7^	1.47 × 10^−5^	9.20 × 10^−5^	5.41 × 10^−10^	1.55 × 10^−7^	9.21 × 10^−5^
Pb	1.43 × 10^−6^	1.79 × 10^−10^	6.42 × 10^−8^	1.50 × 10^−6^	9.08 × 10^−6^	4.46 × 10^−11^	6.42 × 10^−8^	9.14 × 10^−6^
Urban	1.50 × 10^−6^	1.87 × 10^−10^	6.73 × 10^−8^	1.57 × 10^−6^	9.52 × 10^−6^	4.68 × 10^−11^	6.73 × 10^−8^	9.58 × 10^−6^
Rural	1.42 × 10^−6^	1.76 × 10^−10^	6.33 × 10^−8^	1.48 × 10^−6^	8.96 × 10^−6^	4.41 × 10^−11^	6.33 × 10^−8^	9.03 × 10^−6^
Total	1.26 × 10^−4^	4.01 × 10^−4^	3.59 × 10^−6^	5.31 × 10^−4^	8.01 × 10^−4^	1.00 × 10^−4^	3.59 × 10^−6^	9.05 × 10^−4^
Urban	1.23 × 10^−4^	4.21 × 10^−4^	3.05 × 10^−6^	5.47 × 10^−4^	7.80 × 10^−4^	1.05 × 10^−4^	3.05 × 10^−6^	8.89 × 10^−4^
Rural	1.27 × 10^−4^	3.96 × 10^−4^	3.73 × 10^−6^	5.27 × 10^−4^	8.06 × 10^−4^	9.89 × 10^−5^	3.73 × 10^−6^	9.09 × 10^−4^

## Data Availability

The data that support the findings of this study are available from the corresponding author on reasonable request.

## Supplementary Materials

The following supporting information can be downloaded at: https://www.mdpi.com/article/10.3390/toxics11121018/s1, Table S1: The values of the parameters for the noncarcinogenic and carcinogenic risk assessment; Table S2: The reference values for the noncarcinogenic and carcinogenic risk assessment; Table S3: The quality control values of the heavy metals’ measurement [17,46,47,82,83,84,85,86,87,88].
