# Supplementary material for: Health Risk Assessment of Heavy Metals in Indoor Household Dust in Urban and Rural Areas of Chiang Mai and Lamphun Provinces, Thailand
Source: Toxics. 2023 Dec 14;11(12):1018. doi: 10.3390/toxics11121018 (PMC10747779; doi:10.3390/toxics11121018)
Supplement: Supplementary file 1 [file toxics-11-01018-s001.zip › toxics-2694634-supplementary.pdf]

Supplementary Information

Health Risk Assessment of Heavy Metals in Indoor Household Dust in Urban and Rural Areas of Chiang Mai and Lamphun Provinces, Thailand

Kawinwut Somsunun[1,2], Tippawan Prapamontol[1*], Todsabhorn Kuanpun[1], Teetawat Santijitpakdee[1], Kanyapak Kohsuwan[1], Natwasan Jeytawan[1], Nathaporn Thongjan[1]

[1] Research Institute for Health Sciences (RIHES), Chiang Mai University, Chiang Mai 50200, Thailand

[2] PhD Degree Program in Environmental Science, Environmental Science Research Center, Faculty of Science, Chiang University, Chiang Mai 50200, Thailand

Table S1 The values of parameters for non-carcinogenic and carcinogenic risk assessment

Table S2 The reference values for non-carcinogenic and carcinogenic risk assessment

Table S3 Quality control values of heavy metal measurement

*Corresponding author

Tippawan Prapamontol

E-mail: tippawan.prapamontol@cmu.ac.th

Table S1. The values of parameters for non-carcinogenic and carcinogenic risk assessment

Abbrev	Parameter	Value		Unit	Ref
		Adults	Children		
	Non-carcinogenic risk				
ABS	Absorption factor (dermal)	0.001	0.001		Hou et al., 2019[1]
AT	Averaging time	8760	8760	Days	Doyi et al., 2019[2]
BW	Body weight	70	15	kg	
CF	Conversion factor	1.00E-06	1.00E-06	mg kg^{-1}	Isley et al., 2022[3]
ED	Exposure duration	24	6	Years	Hou et al., 2019[1]
EF	Exposure frequency	350	350	Days $year^{-1}$	US EPA[4]
ET	Exposure time	24	24	Hours day^{-1}	US EPA[4]
IngR	Ingestion rate^	100	200	mg day^{-1}	US EPA, 2011[5]
InhR	Inhalation rate	16.3	7.6	m^3 day^{-1}	Hou et al., 2019[1]
PEF	Soil to air particulate emission factor	1.36E+09	1.36E+09	m^3 kg^{-1}	US EPA[4]
SA	Skin surface area	5700	2373	cm^2	Wang et al., 2022[6]
SL	Solid to skin adherence factor	0.07	0.2	mg cm^{-2}	US EPA[4]
	Carcinogenic risk				
24	Hours per day	24	24	hours	
ABS	Absorption factor (dermal)	0.001	0.001		Hou et al., 2019[1]
AT	Averaging time	365*70	365*70	days	US EPA[4]
CF	Conversion factor	1.00E-06	1.00E-06	mg kg^{-1}	Isley et al., 2022[3]
DFS	Age adjusted soil dermal factor	362.4	362.4	mg year kg^{-1} day^{-1}	Doyi et al., 2019[2]
ED	Exposure duration	24	6	years	Hou et al., 2019[1]
EF	Exposure frequency	350	350	Days $year^{-1}$	US EPA[4]
ET	Exposure time	24	24	Hours day^{-1}	US EPA[4]
IR	Intake rate	113	200	mg year kg^{-1} day^{-1}	US EPA[7]
LT	Lifetime	70	70	years	US EPA[4]
PEF	Soil to air particulate emission factor	1.36E+09	1.36E+09	m^3 kg^{-1}	US EPA[4]

Table S2 The reference values for non-carcinogenic and carcinogenic risk assessment

Elements	Non-carcinogenic			Carcinogenic		
	RfD_{ing}	RfD_{inh}	RfD_{der}	SF_{ing}	SF_{inh}	SF_{der}
As	3.00E-04[a]	1.50E-05[a]	1.23E-04[b]	1.50E+00[a]	4.30E-03[a]	1.50E+00[a]
Cd	1.00E-03[c]	5.70E-06[c]	2.50E-05[c]	3.80E-01[c]	7.05E+00[c]	1.52E+01[c]
Cr	3.00E-03[a]	1.00E-04[a]	6.00E-05[b]	5.00E-01[a]	4.20E+01[a]	5.00E-01[a]
Cu	4.00E-02[c]	6.90E-04[b]	1.20E-02[b]	-	-	-
Mn	2.40E-02[a]	5.00E-05[a]	1.84E-03[a]	-	-	-
Ni	2.00E-02[a]	2.06E-02[c]	5.40E-03[b]	1.54E-01[d]	2.60E-04[a]	9.10E-01[a]
Pb	3.50E-03[b]	3.52E-03[c]	5.25E-04[b]	8.50E-03[e]	1.20E-05[d]	2.10E-01[a]
Zn	3.00E-03[a]	3.00E-01[b]	6.00E-02[b]	-	-	-

a: US EPA[4]

b: Ferreira-Baptista and De Miguel., 2005[8]

c: Doyi et al., 2019[2]

d: Wignall et al., 2018[9]

e: OEHHA[10]

Table S3 Quality control values of heavy metal measurement

Metals	LOD (mg kg^{-1})	Standard recovery ± SD (%)	SRM 2584		
			Certified mass fraction (mg kg^{-1})	SRM recovery (%)	%R.S.D
As	2.08	83.93 ± 3.29	17.4 ± 4.2	69.1	8.23
Cd	0.39	93.82 ± 3.25	10.0 ± 1.1	84.7	5.61
Cr	1.11	92.86 ± 4.71	135 ± 9.1	79.7	1.85
Cu	0.58	97.58 ± 3.15	320	90.1	4.55
Mn	0.51	96.98 ± 4.93	370	80.1	1.44
Ni	1.12	81.88 ± 5.84	90	61.5	5.27
Pb	0.60	86.83 ± 7.80	9761	86.5	1.11
Zn	1.54	83.30 ± 5.41	2580 ± 150	81.1	1.53

References

1 Hou, S. *et al.* Pollution characteristics, sources, and health risk assessment of human exposure to Cu, Zn, Cd and Pb pollution in urban street dust across China between 2009 and 2018. *Environment International* **128**, 430-437, doi:https://doi.org/10.1016/j.envint.2019.04.046 (2019).

2 Doyi, I. N. Y., Isley, C. F., Soltani, N. S. & Taylor, M. P. Human exposure and risk associated with trace element concentrations in indoor dust from Australian homes. *Environment International* **133**, 105125, doi:https://doi.org/10.1016/j.envint.2019.105125 (2019).

3 Isley, C. F. *et al.* International Analysis of Sources and Human Health Risk Associated with Trace Metal Contaminants in Residential Indoor Dust. *Environ Sci Technol* **56**, 1053-1068, doi:10.1021/acs.est.1c04494 (2022).

4 EPA, U. *US EPA Regional Screening Levels (RSL) Calculator*, <https://epa-prgs.ornl.gov/cgi-bin/chemicals/csl_search> (

5 EPA, U. (U.S. Environmental Protection Agency, Washington, DC, 2011).

6 Wang, S., Wang, L., Huan, Y., Wang, R. & Liang, T. Concentrations, spatial distribution, sources and environmental health risks of potentially toxic e
lements in urban road dust across China. *Science of The Total Environment* **805**, 150266, doi:https://doi.org/10.1016/j.scitotenv.2021.150266 (2022).

7 US EPA. *Supplemental Guidance for Developing Soil Screening Levels for Superfund Sites*, <https://www.epa.gov/superfund/superfund-soil-screening-guidance> (

8 Ferreira-Baptista, L. & De Miguel, E. Geochemistry and risk assessment of street dust in Luanda, Angola: A tropical urban environment. *Atmospheric Environment* **39**, 4501-4512, doi:https://doi.org/10.1016/j.atmosenv.2005.03.026 (2005).

9 Wignall, J. A. *et al.* Conditional Toxicity Value (CTV) Predictor: An <i>In Silico</i> Approach for Generating Quantitative Risk Estimates for Chemicals. *Environmental Health Perspectives* **126**, 057008, doi:doi:10.1289/EHP2998 (2018).

10 Office of Environmental Health Hazard Assessment (OEHHA). *Technical Support Document for Cancer Potency Factors 2009*, <https://oehha.ca.gov/air/crnr/technical-support-document-cancer-potency-factors-2009> (
